# Animal Heat

**Published:** 1868-07

**Authors:** G. W. Decamp

**Affiliations:** Emery


DENTAL REGISTER.

Vol. XXII.] JULY, 1868. [No. 7.

### Original Communications.

### ANIMAL HEAT.

Read before the Central Ohio Dental Association,

BY DR. G. W. DECAMP.

Gentlemen: There is perhaps no subject which has in times past attracted so much attention as "Heat," and yet we know but little of it; it has baffled the chemist and philosopher so long that it has become the general impression that little or nothing more can be learned of it,—that it is without the grasp of mortals. Our text books state that it is an imponderable; that it is to be found throughout creation and fills vacuity; that it sustains both vegetable and animal life; and without it there could not have been a creation. But when asked, What is Heat? They answer by giving us theories. With "*imponderable*" given as its definition the student is apt to pass it by with the impression that it would be impossible for him to solve the problem.

There have been numerous theories of heat advanced, two of which I shall mention. The first considers heat as a fluid destitute of weight, and capable of flowing from one body to another; the particles of this fluid mutually repelling each other, but are attracted by the particles of other bodies. According to this, the theory of emission, a body cools by losing a part of this fluid. The second theory considers heat as the result of a vibratory motion of the particles of bodies; it is also stated by some authors that this motion is transmitted from one body to another through an elastic fluid, in the same manner that sound is transmitted through the atmosphere. Thus a body in cooling loses a part of this undulatory motion. At the present time the undulatory theory, that "essence of heat is motion," is generally accepted by scientific men.

There are several sources of heat,—the sun, the stars, combustion, heat produced by electricity, magnetism, mechanical powers, and the vital heat generated in animals and vegetables. But it is my intention at this time to present for our consideration, "Animal Heat." One of the most interesting phenomena presented in nature is the power possessed by animals of maintaining a standard temperature independent of the temperature of the surrounding elements.

If we heat a bar of metal to 150° Fah., and expose it to an atmosphere of 55° or 60° Fah., it will immediately part with its heat by radiation and conduction until it becomes of the temperature of the surrounding atmosphere.

With living animals this is not the case; although they part with their heat both by radiation and conduction, yet the common standard temperature is maintained by a continual supply generated in the body.

Many theories as to the manner in which Animal Heat is produced have been presented which within themselves seemed conclusive, when tested have failed. "Thoughts are but dreams till their effects be tried."

In nature one of the great sources of heat is the combustion or oxidation of carbonaceous substances. By experiment it has been found that the rapidity of the oxidation of these substances and the amount of heat generated is in direct proportion to the amount of oxygen admitted, and carbonic acid produced; and whether the combustion be rapid or slow, the same amount of carbonic acid and heat will be produced and oxygen consumed.

There are therefore two general principles upon which the generation of heat seemingly depends, they seem essential to it, and always accompany it. These are the consumption of oxygen and the production of carbonic acid.

These phenomena presented by artificial heat, just referred to, are found in the living body, and as they are here also accompanied by caloric it has led to the supposition that Animal Heat is produced by combustion of carbon.

Levoisier basing his theory upon this supposition, claimed that "Animal Heat" was produced by the direct combination of oxygen and carbon in the pulmonary cells, therefore the lungs were considered the furnace of the body, which eliminated its heat to all parts of the system. If this theory were true, the lungs would be the warmest part of the body, which is not the case, it being proven by experiment that they were not only not warmer than other parts of the body, but on account of their continual exposure and contact with the atmosphere, are cooler. Subsequent experiments also prove that carbonic acid is not formed in the lungs, but exists in the blood before it reaches the lungs.

We have previously stated that in the production of artificial heat the amount of carbonic acid generated, is in direct proportion to the amount of oxygen and carbon consumed, but with animals this law is not valid, which would be the case if the theory of Levoisier were true.

Regnault has found by experiments on animals that the amount of oxygen given off will vary with their food. If it be meat, only about 75 per cent. of the amount absorbed will reappear under the form of carbonic acid, while if fed on vegetable substances over 90 per cent. will be eliminated. In some instances, when the animal was fed exclusively on bread and grain, the proportion was found to be 101 and even 102 per cent. That is, more oxygen was given off in the form of carbonic acid than was taken from the atmosphere; therefore carbonic acid may be under certain circumstances produced in some other way than by direct combustion.

It has also been found that generally a certain amount of oxygen disappears in the body; that there is not as much oxygen eliminated under the form of carbonic acid as is absorbed from the air.

Liebig also advocated the theory of direct combustion as the source of Animal Heat, but differed somewhat, with Lavoisier. He asserted that oxygen was taken into the blood in its passage through the lungs, and that Animal Heat was produced by the oxidation of certain elements of food while circulating in the blood, these substances being converted into carbonic acid and water by the oxidation of their carbon and hydrogen, and eliminated without entering into the formation of any part of the solid tissues. He accordingly divided food into two classes. First, those substances which enter into the formation of the tissues of the body, which embraced albuminous substances, etc. Second, those which were consumed by oxidation, whose only office was to produce Animal Heat. These substances were of the nature of starch. He regarded them as the fuel of the body, taking no part in nutrition.

In considering this subject we must remember that the elimination of Animal Heat is not general as it would be if it were generated by combustion in the blood.

It has been demonstrated by Benard that in the normal condition of the system the blood varies in temperature in different parts of the body. He found it warmer in the portal vein than in the aorta and considerably warmer in the hepatic than in either the portal or vena cava, the blood in the hepatic vein being warmer than that of any other part of the body.

In Professor Yeomans' Chemistry, we find the following in reference to the Cause of Animal Heat. "The union of oxygen with carbon and hydrogen is a source of heat under varied conditions. We can combine them in no way without producing heat. The animal body inhales oxygen and exhales carbonic acid; there has therefore been a union, and that union must have produced heat. Here is a real cause, and one adequate to account for nineteen-twentieths of the heat generated in the human body. Muscular and nervous action produce heat, and this may probably explain the source of the deficiency."

Animal Heat has been placed in special relation to the generation of carbonic acid because in warm-blooded animals respiration is most active, and the products of respiration vary with an increase or diminution of heat; but we find this also true of the excretory products of the body. An increase of the temperature is generally accompanied by an increased activity of the nutritive process, and carbonic acid is not only discharged in greater quantity than usual, but also the elements of urine and perspiration.

It is considered by some of our most scientific medical men, that the inhalation of oxygen and exhalation of carbonic acid, bear the same relation to each other that the food does to ingredients of the urine given off by the kidneys. That "the tissues require a constant supply of solid and liquid food which is introduced through the stomach, and of oxygen which is introduced through the lungs." That "the disintegration and decomposition of the tissues give rise to urea and uric acid, etc., which are discharged with the urine; and also to carbonic acid, which is exhaled through the lungs;" but that "oxygen is not directly converted into carbonic acid, any more than the food is converted directly into the urates."

The numerous decompositions and combinations which are constantly taking place in the body during the nutritive process, doubtless generate internal or Animal Heat. In support of this theory we find that those organs in which these changes are taking place most rapidly generate the greatest amount of heat, etc.

Gentlemen: In this article I have simply presented a few leading theories and hypotheses of scientific men of the past and present; hoping that in comparing and reflecting we may form some opinion as to "what is the truth."

### BAGATELLE.

Editors Register: The reader who will take the trouble to wade through the Dental periodicals for the last six or seven years, will be struck with nothing so much as the multiplicity of views held by Dentists upon subjects about which there should be but one opinion. His labor will afford him much instruction and be productive of some amusement, as well as enable him to set up a criterion by which to judge of the progress which, during the same period, has been made in Dental literature.

Of all the subjects on which different opinions are held, there is none upon which there has been, and is, so much diversity of opinion as on the proper course to be pursued by patients for the preservation of their teeth; and more especially in regard to the use of the brush, and of dentifrices. Some writers recommend the free use of the brush at least once after every meal, and especially at night before going to bed. Others advise, on the contrary, only a very moderate use of the brush, but a very free use of tooth picks and hickory sticks, adding, apologetically for the sticks, that "they form kind tooth brushes." Some, again, who seem to love crotchets for the sake of the reputation of being crotchety, have a hankering after files and silk handkerchiefs. According to these, all deleterious substances between the teeth should be removed with the kerchief, and if there is not space between them for the operation their approximal surfaces should be filed until the requisite space is obtained. Philanthropical persons would benefit such men (if there are any at the present time, and if they are not entirely too hardened to be impressed with religious threats,) by reminding them not passionately, but in a spirit of charity, of the favorite but awful text of the Hardshell Baptist preacher, "*And they shall gnaw a file.*" Hard and soft brushes, again, have each their advocates. A writer, in the first volume of the "*Cosmos*," objects to hard brushes, not only because of their likelihood to irritate the gums, but also because the friction of the hard bristles on the enamel would wear it away,—would, in fact, cut grooves in it, which would eventually, if not plugged, lead to exposure and devitalization of the pulp. You who use hard brushes beware!—half a dozen or more grooves on your central incisors would not be very sightly, even if plugged with gold. The enamel of the teeth is one of the hardest known substances, and is not likely to suffer very material injury from the intermittent friction with a hard brush and water. Such brushing, however, can not take place without. to some extent, involving the gum. On this account it would seem that the soft brush is preferable to the hard; though it must be admitted that there are some mouths so filthy that a soft brush would be comparatively useless to remove the tartar from the teeth.

As regards the frequency with which the brush should be used it is impossible to lay down any rule which shall apply to all cases. Circumstances alone, both for Dentists and patients, can decide the point. There can hardly be a doubt, however, that if the object of the brush is to remove from the inequalities on and around the teeth, all substances which would be likely to induce decay, the brush should be used after each meal; and although the tooth pick is an indispensable companion it is hardly possible that it, alone or in conjunction with hickory sticks, can take the place of the brush.

As regards dentifrices, their constitution seems to be the point about which there is so much difference. Their use is a matter which can only be settled by circumstances, as all Dentists are agreed upon, the chief being whether there is any deposit upon the teeth which can not be removed with the brush and water. A writer in the first volume of the "*Cosmos*" holds, however, that the enamel needs *polishing* sometimes, and the powder must be used, with a "stick of red cedar, orange or hickory wood, about three inches long, wedge shaped, and about one-eighth to one-quarter inch wide." This writer also lays down a rule that the dentifrice should be used not oftener than three times in a month. This is impolitic. Men and women are naturally lazy, and exceptions but prove the rule. When they are sick they have to be coaxed and wheedled into doing what their common sense should teach them they should not hesitate at a moment. Many of the external applications in the shape of liniments, ointments and washes for the relief of pains in the limbs, have little efficacy in themselves. But they are prescribed with the advice that they must be *well rubbed in* with the hand or a woolen cloth. It is the *friction* that is effective in giving relief, and the kind hearted physician has had recourse to a ruse to induce the sufferer to do what, were he acquainted with the real state of the case, he would probably neglect. With nothing are people more neglectful than with their teeth, and like the physician, the kind-hearted Dentist must have recourse to a ruse. The patient must be advised to use, perhaps, a little prepared chalk, at least three times a week, and it must be *well rubbed on with a brush, and plenty of water must be used.*

But it is on the formula of a dentifrice that there is such a diversity of opinion. Castile soap alone is strongly recommended by some, while others, with more reason, recommend it in connection with prepared chalk and some other ingredients of a tonic nature. Nearly every Dentist, however, has his favorite "powder," and, as in most cases, it is compounded by himself, it is not surprising that sometimes articles are found in its composition which are not theoretically admissible, and which are only admitted to "please the customer." When, however, a committee is appointed by a solemn society of Dentists to examine into and report the best formula for a dentifrice, simple minded men are apt to think that the best, or an approximation to it, will be produced. In the "REGISTER" for May it is written in the account of the proceedings of the "Northern Ohio Dental Association," Dr. Charles Buffet, the committee on dentifrices reported the following formula, which the society afterwards adopted·

Pre. Chalk, ℥ vi.; Cuttle Bone, ℥ i.; Peruvian Bark, ℥ ss.; Sugar, ʒ ij.; Castile Soap, ʒ ij.

Now why cuttle bone? Was not prepared chalk sufficient? Cuttle bone when reduced to a fine powder is still somewhat gritty, and *will cut.* If *cutting* is the property required, finely powdered pumice or emery would do as well. Again, one-sixth part of the preparation is composed of sugar—just as much sugar as soap. What useful purpose does this subserve? The object of a dentifrice is to cleanse the teeth, not to supply the patient with sweets; and the ingredients should be entirely innocuous. Sugar is one of those substances which will ferment when brought in contact with decomposing animal matter, such as that found in the saliva, and deposited upon and around unclean teeth. Cane sugar brought in contact with the acids of the mouth, where is the necessary heat and moisture, is liable to Katalytic change and to decomposition, and, according to the circumstances, lactic acid may be generated, or alcohol and carbonic acid. Indeed, from sugar is formed one of the deadliest poisons, oxalic acid. The following table will show the relation between the two:

| | c. | h. | o. | | | c. | h. | o. |
|---|---|---|---|---|---|---|---|---|
| 1 eq. sugar, | 24 | 18 | 8 | } | 12 eq. oxalic acid, | 24 | | 36 |
| 46 eq. oxygen, | | | 36 | } | 18 eq. pts. water, | | 18 | 18 |
| | c. | h. | o. | | | c. | h. | o. |
| | 24 | 18 | 54 | | | 24 | 18 | 54 |

Now when it is considered that there is a large proportion of oxygen in the saliva, one might well hesitate before making sugar one of the ingredients of a dentifrice. To say that it is used to satisfy the sense of taste and to wet the appetite of patients, is a melancholy admission. The writer is acquainted with a Dentist who makes his own "tooth powder," or did make it until he presented *his* formula (which by the way he never followed,) to one of the city druggists. The following dialogue will explain itself:

*Dr.*—Bob, have you put in the sugar?

*Bob.*—Yes sir, I've put in a pound.

*Dr.*—Well put in another pound.

*Bob.*—Won't that be too much sir? its almost one-quarter sugar already.

*Dr.*—No; they like it; they'll lap it up like cats and come back for more.

But perhaps the "committee of one" can give a better reason for his sugar.

EMERY.

